# Sequence-Based Genomic Analysis Reveals Transmission of Antibiotic Resistance and Virulence among Carbapenemase-Producing Klebsiella pneumoniae Strains

**DOI:** 10.1128/msphere.00143-22

**Published:** 2022-05-12

**Authors:** Yi Zhang, Chen Chen, Jing Wu, Jialin Jin, Tao Xu, Yang Zhou, Peng Cui, Jiazhen Chen, Shu Chen, Ning Jiang, Wenhong Zhang

**Affiliations:** a Department of Infectious Diseases, National Medical Center for Infectious Diseases, Shanghai Key Laboratory of Infectious Diseases and Biosafety Emergency Response, Huashan Hospital, Shanghai Medical College, Fudan University, Shanghai, China; b State Key Laboratory of Genetic Engineering and Institute of Biostatistics, School of Life Sciences, Fudan University, Shanghai, China; JMI Laboratories

**Keywords:** *Klebsiella pneumoniae*, transmission, evolution, hypervirulence

## Abstract

Carbapenemase-producing Klebsiella pneumoniae (CP-Kpn) are a major concern for nosocomial infections. We previously reported an intensive care unit (ICU) outbreak of CP-Kpn. This study investigated the transmission pattern and genetic characteristic of CP-Kpn in the hospital during the outbreak period. Whole-genome sequencing was retrospectively performed on 173 CP-Kpn isolates. Pairwise single-nucleotide polymorphism (SNP) distances were calculated to determine SNP thresholds for clustering. Plasmids and mobile genome elements (MGEs) were identified through short- and long-read sequencing. Strains were classified into three groups, sequence type 11 (ST11) (86.12%), ST15 (9.83%), and other ST. An SNP threshold of 16 revealed a 66.47% clustering rate. ICU admission and meropenem use proportions were significantly higher in clustered patients than in unique patients. MGE distribution was consistent with the phylogenetic tree. Of the isolates, 53.18% were CP-Kpn with hypervirulence genes. We identified five plasmids carrying virulence genes, and four of them have not been previously reported. Clonal transmission was the main cause of CP-Kpn infections in the hospital. Multidrug resistance genes and MGE variations were correlated with clustering. Finally, four novel plasmids carrying virulence genes were identified. The findings highlight the control of CR-Kpn transmission through prevention measures to reduce nosocomial infections.

**IMPORTANCE** In this study, we combined genomic and epidemiological analyses and defined an optimal cutoff value for SNP difference that could be used to aid investigation in tertiary hospital in China. We revealed clonal transmission was the main cause of CP-Kpn infections in the hospital and identified four novel plasmids carrying virulence genes. Our results strongly suggested that dominant CP K. pneumoniae strains lead to outbreaks and described different evolutionary patterns of plasmids carrying multidrug resistance and virulence genes.

## INTRODUCTION

Carbapenemase-producing Klebsiella pneumoniae (CP-Kpn) refers to carbapenem-resistant K. pneumoniae that produces carbapenemases (1). Unlike the dissemination of CP-Kpn strain sequence type 258 (ST258) in United States, the majority of CP-Kpn in China are of the CP-Kpn ST11 clone (2). Genomic evidence has confirmed the high prevalence of CP-Kpn in Europe is driven by nosocomial infections of clustered cases. Moreover, Klebsiella are common bacterial hosts for dissemination of mobile genome elements (MGEs) between, and even across, species (3). MGE-mediated drug resistance makes hospital-acquired infections difficult to prevent and control (4, 5). In China, despite CP-Kpn outbreaks being reported, the transmission pattern based on epidemiologic link in hospitals remains unclear.

In addition to threats from CP-Kpn, hypervirulent strains of K. pneumoniae have also emerged over the past 4 decades. The hypervirulent strains are usually isolated from community-acquired infections and may cause liver abscess, bloodstream infection, or meningitis, among other pathological conditions (6, 7). The reported best-characterized virulence factors with experimental support for conveying the hypervirulent phenotype, including *iuc*, *iro*, *rmpA*, and *rmpA2*, are encoded by genes present on hypervirulent plasmids (7). Multidrug-resistant (MDR) clones might bring a threat to the convergence of hypervirulence and multidrug resistance because they are more likely to acquire virulence genes than hypervirulent clones are to acquire resistance genes (8). Virulence gene carriage has been reported to be 34.2% for carbapenem-resistant K. pneumoniae in China (9), which is a public health concern.

We reported a CP-Kpn outbreak in an intensive care unit (ICU) and tracked the outbreak using whole-genome sequencing (WGS) (10). In the current study, we expand our research by performing a genomic investigation on other strains collected during the outbreak from other patients in the same hospital.

## RESULTS

### Enrolled patient characteristics and corresponding isolates.

A total of 173 CP-Kpn isolates were collected from 136 patients from December 2016 to April 2017 at Huashan Hospital. As shown in Table 1, most CP-Kpn isolates were cultured from sputum samples (102), with the remaining isolates coming from urine samples (35), focal specimens (15), blood cultures (10), cerebrospinal fluid (CSF) samples (5), catheters (3), pharyngeal swabs (2), and bronchoalveolar fluid (BALF) samples (1). As a tertiary hospital in Shanghai, China, some patients (67/136, 49.26%) were transferred from referring or other hospitals. Among the patients included in the study, 72 (52.94%) were admitted with ICU care and 64 (47.06%) with non-ICU care. We identified infection or colonization through both clinical manifestations and laboratory examinations (computed tomography examinations) and found 80.88% (110/136) of them were infected and 19.12% (26/136) have CP-Kpn colonization. Overall, 114 (80.88%) patients survived and 22 (16.18%) patients died.

**TABLE 1:** Baseline characteristics of enrolled patients and isolates

Characteristic	No. (%)
Patient	
Sex	
Male (*n*)	102 (75.00)
Female (*n*)	34 (25.00)
Age (yr)	
<30	10 (27.78)
30–60	72 (41.62)
>60	54 (31.21)
Transferred from referring hospital	67 (49.26)
Clinical outcome	
Death	22 (16.18)
Relieved	114 (83.82)
Isolate	
Ward distribution	
ICU-1	31 (17.92)
ICU-2	29 (16.76)
ICU-3	8 (4.62)
Other ICUs	4 (2.31)
Non-ICUs	
Department of Geriatrics	15 (8.67)
Department of Endocrine	2 (1.16)
Department of Respiratory	3 (1.73)
Department of General Surgery	6 (3.47)
Department of Outpatient and Emergency	10 (5.78)
Department of Infectious Diseases and Antibiotics	13 (7.51)
Department of Neurosurgery	3 (1.73)
Other^*a*^	11 (6.36)
Source	
Sputum	102 (58.96)
Urine	35 (20.23)
Blood	10 (5.78)
CSF	5 (2.89)
Focal specimens^*b*^	15 (8.67)
Pharyngeal swab	2 (1.16)
Catheter	3 (1.73)
BALF	1 (0.58)

a Other here means gastroenterology, hand surgery, rehabilitation, and special wards.

b Focal specimens include bile, secretion, ascites, punctuation fluid, and tissue.

Basic genomic information of the isolates was obtained from WGS analysis. The vast majority of isolates (149/173, 86%) were classified as ST11, and 17 (9.8%) were identified as ST15. The remaining isolates included ST1, ST23, ST278, ST290, ST437, and ST719. The ST11 isolates included 125 of capsular type KL64-wzi64, 16 of capsular type KL47-wzi47, and 3 strains of unclear KL capsular type.

### Clonal transmission and clustering factors of CP-Kpn isolates.

**FIG 1:**
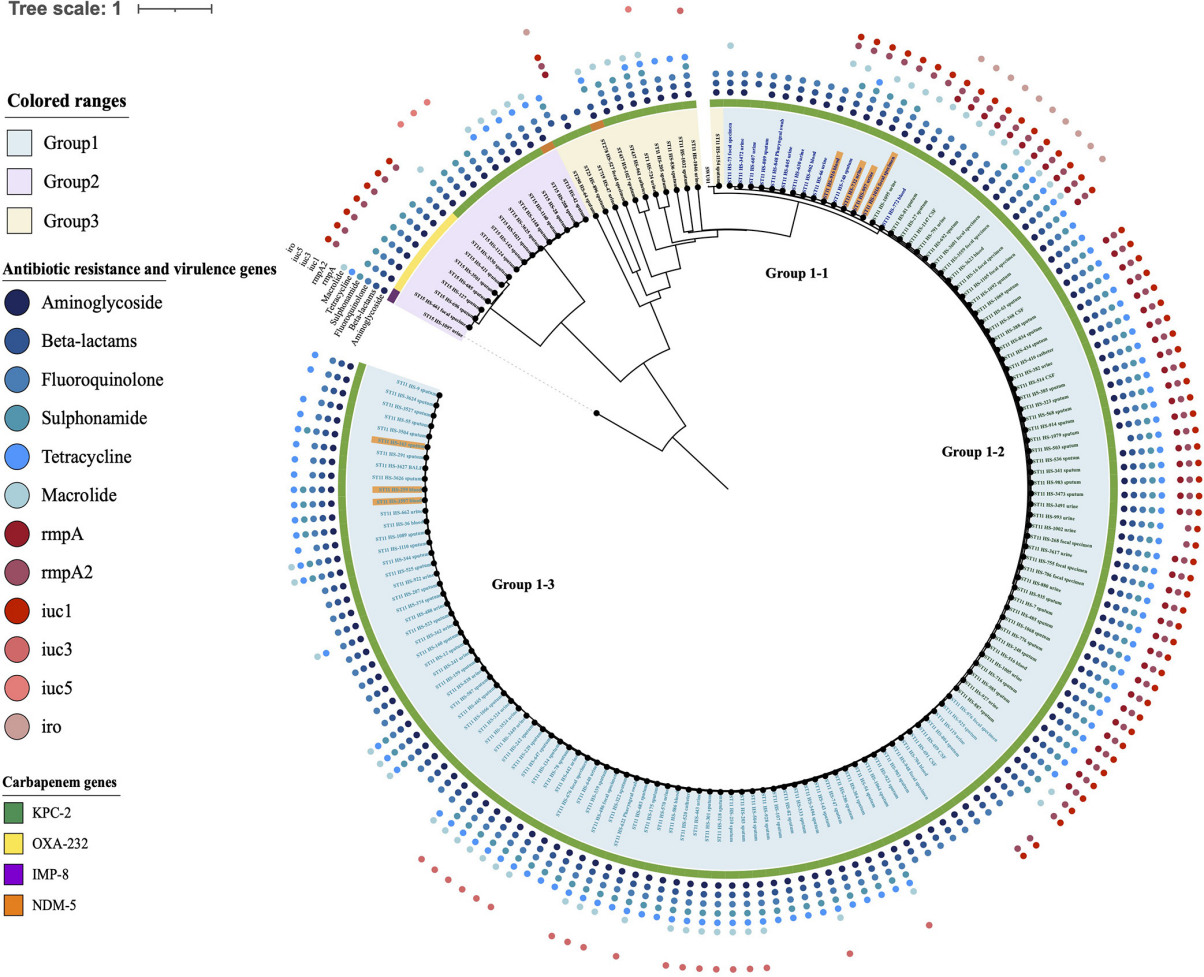
Phylogenetic tree of collected CP-kpn strains. The blue, purple, and yellow color range shows groups 1, 2, and 3. In group 1, the dark blue, green, and light blue text indicates group 1-1, 1-2, and 1-3. For carbapenem genes, KPC-2, OXA-232, IMP-8, and NDM-5 are represented by green, yellow, purple, and orange stripes. The circles outside the tree illustrate the resistance genes (blue circles) and virulence genes (red circles). The three orange-labeled isolates in group 1-3 are outbreak cluster 1 isolates, and the four orange-labeled isolates in group 1-1 are outbreak cluster 2 isolates.

The evolutionary relationships of the 173 CP-Kpn isolates are presented in Fig. 1. The phylogenetic tree was categorized into group 1 (144 isolates), group 2 (17 isolates), and group 3 (12 isolates). We have reported two clustered CP-Kpn outbreaks; the first cluster included three isolates of group 1-3, and the second cluster included four isolates in group 1-1.

**FIG 2:**
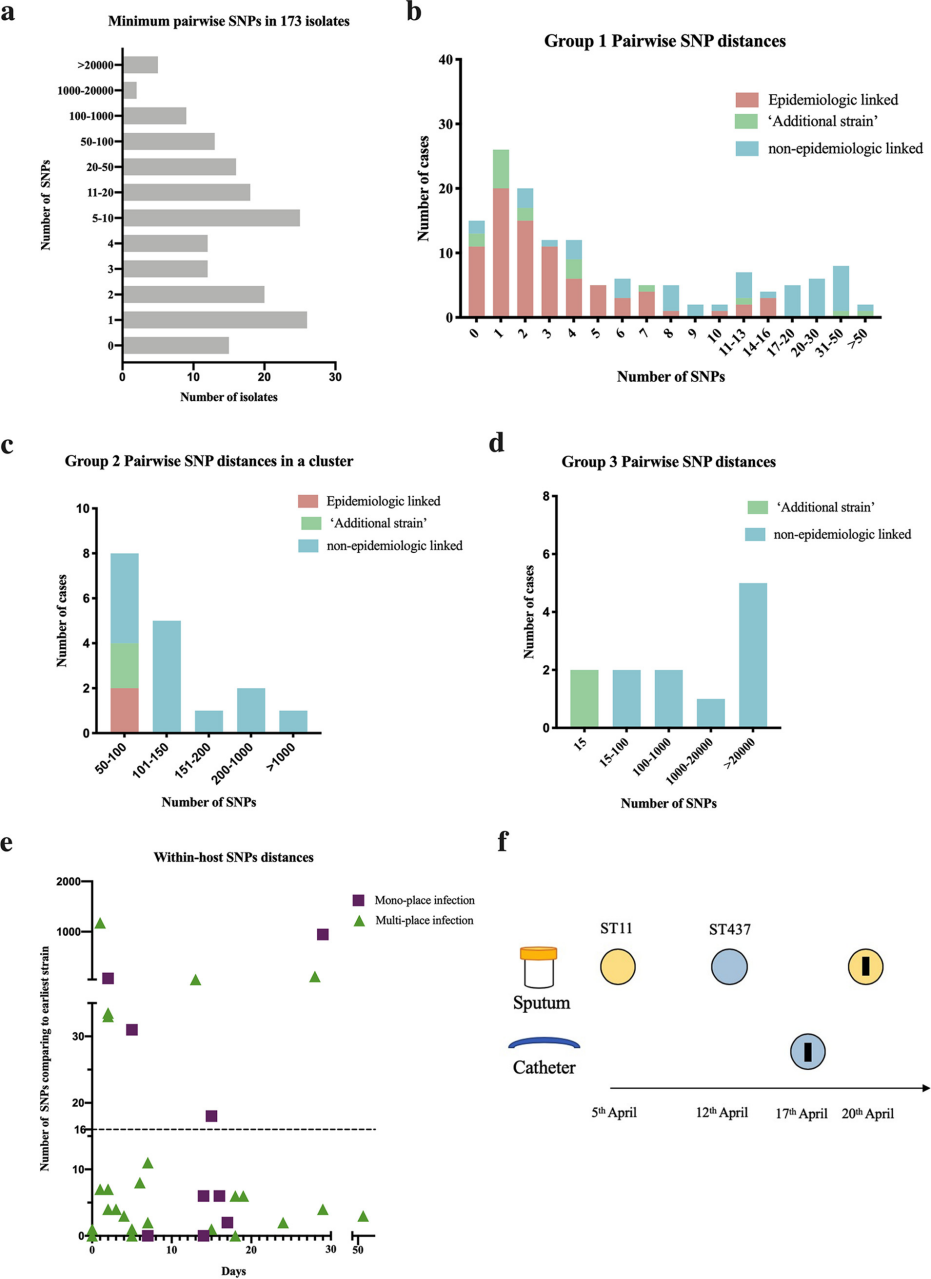
Pairwise SNP distances in 173 CP-kpn strains, clustering distribution, and within-host SNP distances in patients who contributed more CP-kpn strains. (a) Pairwise SNP distances in 173 isolates. (b to d) Minimum SNP distances in isolates of groups 1, 2, and 3. Red shows strains with epidemiologic link with the closest strain, while blue shows strains without epidemiologic link with the closest strain. The green one shows an independent strain (a branch formed by multiple strains isolated from the same patient without other CP K. pneumoniae strains). (e) Distribution of within-host SNP distance (SNP distance compared to the earliest isolated strain) by day. (f) The evolution dynamics of strains in patient 28. The black stripe means one SNP.

Calculation of the minimum pairwise SNPs revealed 110 of the 173 isolates (63.58%) differed by no more than 10 SNPs (Fig. 2a). Epidemiologic investigations identified epidemiologic links in 84 of the 173 CP-Kpn isolates (48.55%). As shown in Fig. 2b to d, patients lacking epidemiologic links were separated by 16 SNPs. Therefore, we defined a CP-Kpn genomic cluster as differing by no more than 16 SNPs. Accordingly, the 173 isolates were classified into 14 clusters, ranging in size from 2 to 32 isolates. We found 6 strains from 2 patients in group 1 as additional strains. The clustering rate of groups 1, 2, and 3 was 83.3% (115/138), 0.00% (0/17), and 0.00% (0/10), respectively, indicating group 1 was the dominant group of CP-Kpn strains in the hospital. This also demonstrated that clonal transmission was the main cause of CP-Kpn nosocomial infections in the hospital.

We then analyzed the clinical characteristics, laboratory test results, and outcomes between the 90 clustered patients and 44 unique patients (Table 2). The proportion of ICU admissions in the clustered patients (61.11%) was significantly higher than that of the unique patients (36.36%; *P* = 0.007). Furthermore, the clustered patients were prescribed meropenem before isolation of CP-Kpn at a higher percentage than that of the unique patients (41.11% versus 20.45%, *P* = 0.018). C-reactive protein levels in the clustered groups were relatively higher than that in the unique patients (54 versus 33.85, *P* = 0.046). Finally, the death rate on discharge of patients infected with dominant CP-Kpn strains was significantly higher than that of the remaining patients (22.22% versus 4.55%, *P* = 0.019).

**TABLE 2:** The clinical factors and outcome in clustered and unique patients

Parameter	Value(s) for:		*P* value^*a*^
	Clustered （90 patients）	Unique (44 patients)	
Transferred from referring hospital [% (no./total no.)]	51.11 (46/90)	45.45 (20/44)	0.539
Length of stay, in median (range) days	24 (3–863)	25 (5–57)	0.761
Admission to ICU [% (no./total no.)]	61.11 (55/90)	36.36 (16/44)	0.007*
History of invasive operation [% (no./total no.)]	43.33 (39/90)	34.09 (15/44)	0.306
Recent surgery before isolation [% (no./total no.)]	13.33 (12/90)	18.18 (8/44)	0.460
History of meropenem use before positive isolation [% (no./total no.)]	41.11 (37/90)	20.45 (9/44)	0.018*
Mortality [% (no./total no.)]	22.22 (20/90)	4.55 (2/44)	0.019*
Laboratory tests [median (range)]			
WBC, 10^9^/liter	9.87 (3.84–33.73)	8.58 (1.31–23.99)	0.410
C-reaction protein, mg/L	54 (3.11–210)	33.85 (3.11–106)	0.046
Procalcitonin, ng/mL	0.35 (0.05–46.1)	0.42 (0.06–3.95)	0.957

a *, *P* < 0.05.

Several phylogenetic trees of confirmed clusters of CP-Kpn isolates from ICUs were generated, a significant factor in forming clusters. ICU-1, -2, and -3 had 30, 20, and 17 beds, respectively. Isolates from ICU-1 and ICU-2 clusters shared high similarity with fewer than 30 SNPs among more than 10 CP-Kpn isolates. In addition, isolates from the Department of Geriatrics similarly exhibited this phenomenon, in that only 17 SNPs existed among 9 CP-Kpn isolates. In comparison, the group 3 cluster consisted of 6 ST15 strains carrying *OXA-232* with a total of 150 SNPs.

Next, we carried out analyses of patients who contributed more than one CP-Kpn isolate to distinguish within-host evolution or mixed infection of clustering. A total of 63 isolates from 28 patients were included in the analysis, for which we calculated SNP distance of the earliest isolate from each patient (Fig. 2e). For monosource isolates, we classified six patients as within-host infections and three patients as mixed infection (see Table S1 in the supplemental material). Patient 28 was found to have both a mixed infection and within-host evolution of CP-Kpn (Fig. 2f).

10.1128/msphere.00143-22.1 TABLE S1 Analysis of patients with more than one CP-Kpn isolate. Download Table S1, DOCX file, 0.02 MB. Copyright © 2022 Zhang et al. 2022 Zhang et al. https://creativecommons.org/licenses/by/4.0/ This content is distributed under the terms of the Creative Commons Attribution 4.0 International license.

### CP-Kpn isolate phenotypic and genomic resistance and virulence profiles.

We first conducted phenotypic antibiotic susceptibility tests (AST) of the CP-Kpn isolates. All the isolates were found to be multidrug resistant according to AST results (Table S2). The carbapenem resistance genes included *KPC*-2 (163), *OXA-232* (7), *NDM-5* (2), and *IMP-8* (1). Coverage analysis of common extended-spectrum beta-lactamases (ESBLs) and broad-spectrum beta-lactamase genes indicated a stable structure of inheritance through transmission (Fig. S1).

10.1128/msphere.00143-22.2 TABLE S2 Antimicrobial resistance profile of traditional AST and whole-genome sequencing-based identification. Download Table S2, DOCX file, 0.02 MB. Copyright © 2022 Zhang et al. 2022 Zhang et al. https://creativecommons.org/licenses/by/4.0/ This content is distributed under the terms of the Creative Commons Attribution 4.0 International license.

10.1128/msphere.00143-22.5 FIG S1 Coverage of ESBL genes in enrolled CP-Kpn strains. Download FIG S1, TIF file, 1.9 MB. Copyright © 2022 Zhang et al. 2022 Zhang et al. https://creativecommons.org/licenses/by/4.0/ This content is distributed under the terms of the Creative Commons Attribution 4.0 International license.

A high carriage rate of virulence gene was also detected. In total, 92 of the 173 isolates exhibited high virulence scores (≥4) according to Kleborate (Table S3). The virulence gene *rmpA* was present in 48 of the isolates that also carried *rmpA2*. Twenty additional isolates were identified to only carry *rmpA2*. Only the ST23 isolate (HS-896) had *colibactin* (*clb*). Meanwhile, *aerobactin 1* (*iuc1*) was present in 39.31% (68/173) of the isolates, and all strains possessed *rmpA2*. *iuc3* and *iuc5* were detected in 20 and 4 isolates, respectively. *Salmochelin 1* (*iro1*) was found to exist in 11 isolates that at the same time also had *rmpA*, *rmpA2*, *iuc1*, and *iro1*.

10.1128/msphere.00143-22.3 TABLE S3 The virulence score ranges, from 0 to 5. Download Table S3, DOCX file, 0.01 MB. Copyright © 2022 Zhang et al. 2022 Zhang et al. https://creativecommons.org/licenses/by/4.0/ This content is distributed under the terms of the Creative Commons Attribution 4.0 International license.

### Comparative analyses of plasmids carrying antibiotic resistance and MGEs.

Although the dominant group 1 shared a close core genome relationship among 144 isolates, accessory genome mapping identified three clades of group 1, including group 1-1, group 1-2, and group 1-3. Based on 13 isolates that underwent short-read and long-read hybrid assembly (Table S4), we found plasmids carrying multidrug resistance genes varied rapidly through transmission and correlated with the clustering.

10.1128/msphere.00143-22.4 TABLE S4 List of isolates sequenced by Illumina and Nanopore and aligned plasmid. Download Table S4, DOCX file, 0.02 MB. Copyright © 2022 Zhang et al. 2022 Zhang et al. https://creativecommons.org/licenses/by/4.0/ This content is distributed under the terms of the Creative Commons Attribution 4.0 International license.

**FIG 3:**
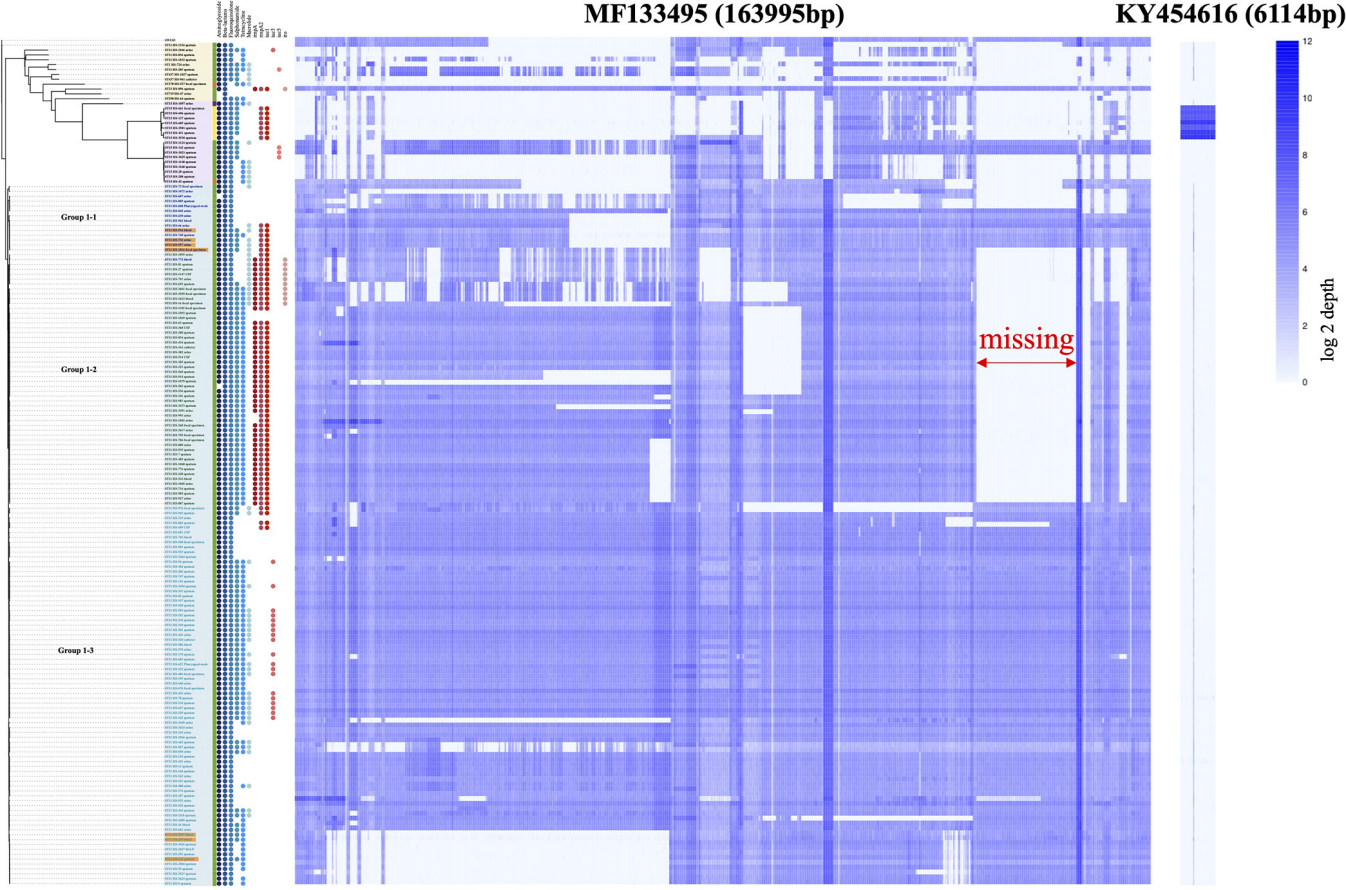
Alignment of plasmids carrying KPC-2 (MF133495; a 163,996-bp plasmid) and OXA-232 (KY454616; a 6,114-bp plasmid). The blue bar shows log_2_ depth.

The mapping coverage of plasmid MF133495.1 in 146 isolates exceeded 60% (Fig. 3). The distribution and mapping region were classified into three groups, consistent with groups 1-1, 1-2, and 1-3 in the phylogenetic tree. We observed that a 130,540- to 149,540-bp region of MF133495.1 was missing from group 1-2.

A total of seven ST15 OXA-232 isolates had a 99.98% alignment ratio with plasmid KY454616.1, a 6,141-bp plasmid. The plasmids carrying NDM-5 in HS-527 and IMP-8 in HS-1097 were also identified (Table S4). Among the plasmids with other multidrug resistance genes, plasmid MF133496.1 was the most common, with which 75 of the isolates reached more than 90.00% coverage.

Following the preliminary alignment, we identified 17 MGEs, including insertion sequences (IS), transposons (Tn), origins of transfer (oriT), and integrative and conjugative elements (ICEs). Among the IS, ISkpn1, ISkpn14, and ISkpn26 exhibited a high carriage rate and copy number in CP-Kpn strains, and variations were found to be in accordance with the clustering (Fig. S2). Transposon Tn*1721* was covered more in group 1-1 than that in group 1-2. As shown in Fig. S3, the variance and mapped regions of oriT and ICE were in accordance with the clustering groups.

10.1128/msphere.00143-22.6 FIG S2 Alignment of insertion sequences and transposon 1721. The 11 insertion sequences are IS*K*pn1, IS*K*pn6, IS*K*pn14, IS*K*pn18, IS*K*pn19, IS*K*pn26, IS*K*pn27, IS*K*pn28, IS*K*pn33, IS*K*pn34, and IS*K*pn38. Download FIG S2, TIF file, 1.1 MB. Copyright © 2022 Zhang et al. 2022 Zhang et al. https://creativecommons.org/licenses/by/4.0/ This content is distributed under the terms of the Creative Commons Attribution 4.0 International license.

10.1128/msphere.00143-22.7 FIG S3 Alignment of *oriT* and ICEs in enrolled CP K. pneumoniae strains. Download FIG S3, TIF file, 1.0 MB. Copyright © 2022 Zhang et al. 2022 Zhang et al. https://creativecommons.org/licenses/by/4.0/ This content is distributed under the terms of the Creative Commons Attribution 4.0 International license.

### Comparative analysis of plasmids carrying virulence genes.

**FIG 4:**
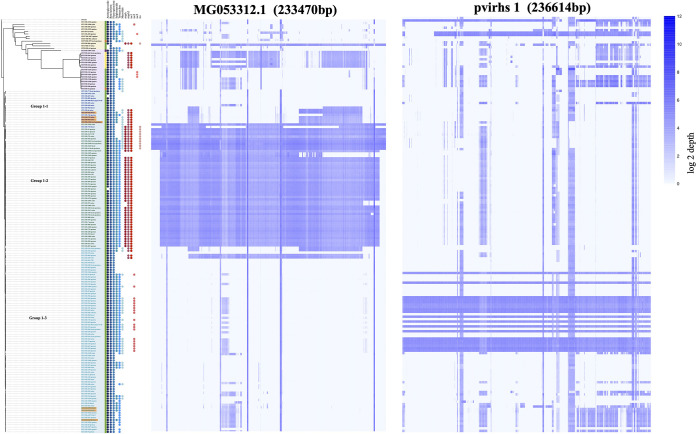
Alignment of plasmids carrying virulence genes. (Left) Plasmid MG053312.1 (233,470 bp). (Right) pvirhs1 (236,614 bp). The blue bar shows log_2_ depth.

Based on mapping coverage, when the alignment coverage was higher than 85%, a virulence plasmid was expected to exist. We identified five plasmids carrying virulence genes, of which four were not previously reported. MG053312.1 plasmid was detected in 47 ST11 isolates in group 1-2 and one ST23 isolate in group 3 and had mapping coverages ranging from 93.60% to 100.00% (Fig. 4 and Fig. S4).

10.1128/msphere.00143-22.8 FIG S4 Coverage of plasmids carrying carbapenem genes, virulence genes, and other multidrug resistance genes. Download FIG S4, TIF file, 1.2 MB. Copyright © 2022 Zhang et al. 2022 Zhang et al. https://creativecommons.org/licenses/by/4.0/ This content is distributed under the terms of the Creative Commons Attribution 4.0 International license.

We found 20 ST11 isolates in group 1-3 that carried pvirhs1 with *iuc3* and several antibiotic resistance genes. In contrast to plasmid MG053312.1, the presence of pvirhs1 had no relationship with core genome distances. Isolates HS-3494, HS-107, and HS-333 shared 0 SNPs, and pvirhs1 was only present in HS-3494. As for the ST15 isolates with high virulence, seven possessed pvirhs2 with *iuc1* and *rmpA2* and three were confirmed to carry pvirhs3 with *iuc5*. No antibiotic resistance genes were detected in pvirhs2 or plasmid MG053312.1. A total of 8 strains carried pvirhs4 (Table 3). Whether the remaining 6 isolates with high virulence scores had plasmids containing virulence genes was not determined and needs to be investigated in a future study.

**TABLE 3:** Features of plasmids carrying virulence genes in our study

Name	No.	Coverage (%)	Length (bp）	Virulence gene(s)	Antibiotic resistance genes	ST
pvirhs1	20	95.80–100.00	238,515^*a*^	*iuc3*	*aadA1*, *mefB*, *cmlA1*, *sulIII*, *dfrA12*	ST11
pvirhs2	7	100.00	154,384^*b*^	*iuc1*, *rmpA2*		ST15
pvihs3	3	100.00	130,295^*c*^	*iuc5*	*aadA22*, *cmlA5*, *catA2*	ST15
pvirhs4	8	85.50–100.00	314,550^*d*^	*iuc1*, *rmpA2*	*aac3-IId*, *armA*, *qnrB4*, *msrE*, *mphA*, *mphE*, *sulI*, *DHA-1*, *SHV-12*, *TEM-30*	ST11
MG053312.1	48	93.60–100.00	233,470	*iuc1*, *iro1*, *rmpA*, *rmpA2*		ST11 (*n* = 47), ST23 (*n* = 1)
Not identified	6					

a Reference plasmid HS-3494.

b Reference plasmid HS-3501.

c Reference plasmid HS-142.

d Reference plasmid HS-732.

## DISCUSSION

The emergence and expansion of CP-Kpn has resulted in a bottleneck in effective antimicrobial treatment. Using molecularly based diagnostic techniques, hospitals are seeing increased transmission of CP-Kpn with multiantibiotic resistance and hypervirulence, which is promoted through recombination and conjunction of MGEs, especially plasmids (9).

Here, we performed a retrospective epidemiologic and genomic study and observed that CP-Kpn ST11 KPC-2 accounted for the majority of the isolated strains, with most of them belonging to KL64-wzi64, which is reported to survive longer and may correlate with higher virulence (11). It is noted that ST15 strains have been rarely reported in hospitals in China. KPC-2 was reported to be the most common carbapenemase type in K. pneumoniae (76.5%) from a large multicenter study in China, which is consistent with our studies. The ST15 K. pneumoniae and OXA-48 family genes were rare in China (2.6%, 0.1%). Here, we found a higher proportion of ST15 K. pneumoniae and OXA-48 family detection (9.83%, 4.05%) that might be due to hospital-acquired transmission (12). For hypervirulent strains, we detected a relatively high proportion of isolates that exhibited high virulence scores, similar to previous studies (9). The virulence of these isolates needs to be confirmed *in vivo* and *in vitro* in future studies.

Phylogenetic analysis indicated close relationships between isolate HS-3504 from the Department of Emergency and the reported index strain (HS-3527) of cluster 1. As previously reported by several researchers, CP-Kpn outbreaks commonly occur in ICUs, which forces care bundles in ICUs to be established and surveilled (13, 14).

In the current study, we defined an optimal cutoff value of 16 for SNP differences to be used in aiding investigations in tertiary hospitals in China. Previously, David et al. focused on ST258/512, which is the most common clone in Europe, and proposed 21 SNPs as optimal to discriminate hospital clusters (15). Meanwhile, an SNP threshold of 11 was empirically used to classify clusters in hospitals in the United States (16). In our study, we combined epidemic information and SNP as previously described (17) and achieved a suitable cutoff for ST11 isolates in tertiary hospitals in China, which may be widely helpful for future epidemiologic investigations.

Using genomic analysis, we depicted the dynamics of MGEs through transmission, all of which were consistent with the phylogenetic clustering. Among the missing fragments, we identified plasmid SOS inhibition protein A (*psiA*), *psiB*, antirestriction protein *ardA*, methylase, plasmid partition protein ParA (*parA*), and Tn*3*. Plasmid incompatibility is closely correlated with plasmid partition, and it is reported that antirestriction and antimodification systems prevent foreign DNA from entering a bacterial host (18). After annotation, the upstream region of the missing fragment included IS*26*, while the downstream region included ISkpn26 (Fig. S5).

10.1128/msphere.00143-22.9 FIG S5 Upstream and downstream structure of missing part in plasmid MF133495 in group 1-2. Download FIG S5, TIF file, 2.5 MB. Copyright © 2022 Zhang et al. 2022 Zhang et al. https://creativecommons.org/licenses/by/4.0/ This content is distributed under the terms of the Creative Commons Attribution 4.0 International license.

It has been previously shown that the hypervirulence of K. pneumoniae is generally associated with a typical virulence plasmid of approximately 200 kb (19). In our current study, we identified four novel plasmids carrying virulence genes. The existence of STs of pvirhs1 and pvirhs4 plasmids indicated the acquisition of plasmids carrying virulence genes correlates with CP-Kpn strain genetic backgrounds. The evolution process of strain convergence for carbapenem resistance and virulence included potential two paths. Either an original hypervirulent strain acquired an MDR plasmid or a CP-Kpn strain acquired a virulence plasmid (19). It is noted that plasmid MG053312.1 was first identified in an ST11-KPC2 K. pneumoniae strain (20). Furthermore, we found its coexistence in both ST11 and ST23 strains, which indicated this plasmid potentially originated from ST23 hypervirulent strains.

There were several limitations in our study. First, it was strictly a retrospective analysis of CP-Kpn isolates collected during an outbreak and represented a relatively short period to analyze variations in CP-Kpn throughout the hospital. Second, the cutoff value of 16 SNPs has not been validated. This needs to be confirmed in the future using a mathematical model or a larger data set of CP-Kpn strains. Finally, the level of virulence conferred by the plasmids carrying virulence genes has not been confirmed *in vitro* or *in vivo*, which should be done in subsequent studies.

### Conclusions.

Our findings demonstrated that clonal transmission was the main cause of CP-Kpn nosocomial infections in this Chinese tertiary hospital. Multidrug resistance genes and MGEs varied rapidly through transmission and correlated with clustering, and novel plasmids carrying virulence genes were identified. The findings call for more prevention measures for transmissions to reduce CP-Kpn nosocomial infections.

## MATERIALS AND METHODS

### Patients’ characteristics and corresponding isolates.

We conducted a retrospective study to analyze CP-Kpn isolates collected from December 2016 to April 2017 at Huashan Hospital, with more than 1,200 beds. Carbapenem resistance strains were identified by inhibition zones of imipenem or meropenem smaller than 19 mm in diameter, and after WGS, we enrolled strains carrying carbapenemase genes for analysis. Nine strains without carbapenem genes were excluded from the study. Isolates collected from the same sites in the same patient with accordant antibiotic susceptibility tests were also excluded from the study. The isolates had been cultured from clinical samples obtained from patients admitted to Huashan Hospital during this period. A total of 173 isolates were included in the study.

Antibiotic susceptibility test results were collected and analyzed using the Clinical and Laboratory Standards Institute version 2017 guidelines. Synchronous clinical data and laboratory information were also recorded. Ethical approval was achieved from Huashan Hospital Committee (2019429). Signed informed consent of the patients was obtained.

### WGS and plasmid confirmation.

The isolates were recovered on LB medium from stored clinical isolates kept in a −80°C freezer. A single colony was selected to prepare for WGS. Qualified DNA libraries were sequenced on an Illumina Nova-seq platform (Illumina, USA) using a pair-end 150-bp strategy. *De novo* assembly of short-read sequencing data was performed using SPAdes (v3.11.1) with default parameters. Sequence type, antibiotic resistance genes, virulence genes, virulence loci, K and O types, and virulence scores were further confirmed using Kleborate (https://github.com/katholt/Kleborate). Plasmid multilocus sequencing types were identified through the pMLST 1.4 database as previously reported (10). Detected K. pneumoniae aerobactin synthesis loci (*iuc*) were classified as lineage *iuc1*, *iuc3*, and *iuc5* according to previous reports (21). For better assembly and interpretation of plasmid variation, 13 isolates were sequenced at 1 to 2 GB per isolate on a Nanopore GridION platform (Oxford, United Kingdom). Average depth was more than 300×, and the error rate ranged from 10 to 15%. Unicycler (22) (v 0.4.7) was used to assemble the Illumina short reads and Nanopore long reads. To characterize the plasmids, we performed alignment for the sequences with reported K. pneumoniae plasmid sequences from the National Center for Biotechnology Information (NCBI). Plasmids were annotated using the Rapid Annotations using Subsystems Technology (RAST) annotation system (https://rast.nmpdr.org).

### SNP analysis and phylogenetic tree construction.

K. pneumoniae strain SWU01 (GenBank accession no. CP018454.1) was used as the reference genome for WGS read mapping. Bowtie2 (v 2.3.3.1) (23) was used for mapping reads, and candidate SNPs were identified using SAMtools (v 1.9). We constructed phylogenetic trees from 81,081 vertically inherited SNPs in all 173 isolate sequences using Molecular Evolutionary Genetics Analysis (MEGA) X (24) with maximum-likelihood estimation and the general time-reversible (GTR)+γ+I nucleotide substitution model suggested by jModelTest (v2.1.10) (25). The results were used to annotate the phylogenetic tree in iTOL v4 (26) (https://itol.embl.de/index.shtml).

### Pairwise SNP calculation and cluster definition.

We first calculated the core chromosomal number of pairwise SNP distances. The minimum numbers of SNPs of the most closely related isolates were identified for each isolate in this study. Epidemiologic link was defined as overlapping time in the same room during hospitalization. An SNP cutoff was determined and combined with the epidemiologic link to aid in defining clusters and investigating the outbreak, as previously described (15, 17, 27, 28). A branch formed by multiple strains isolated from the same patient without other CP-Kpn strains was not considered a cluster. These strains were defined as an additional strain in this study to aid in classification. Therefore, combined with the epidemiologic link, patients were classified into three categories: with epidemiologic link, nonepidemiologic link, and additional strain.

### Comparative analysis of MGEs.

To determine the dynamics of antibiotic resistance and virulence associated with accessory genome, we analyzed plasmids that had been confirmed by a combination of short-read and long-read sequencing. After the preliminary alignment, we identified 17 MGEs to perform comparative alignment. The MGEs included insertion sequence IS*K*pn1, IS*K*pn6, IS*K*pn14, IS*K*pn18, IS*K*pn19, IS*K*pn26, IS*K*pn27, IS*K*pn28, IS*K*pn33, IS*K*pn34, and IS*K*pn38, Tn*1721*, origin of transfer (oriT) oriT_pEC958 and oriT_R100, and ICEKp1, ICEKpnHS11286-1, and ICEKpnHS11286-2.

### Statistical analysis.

Patient characteristics and laboratory examinations as continuous variants were described by means when they conformed to the Kolmogorov-Smirnov test and by medians when they failed to conform. Pearson’s chi-square test was used to evaluate independent binomial variables. We performed analysis of variance (ANOVA) when normal distribution was satisfied and Kruskal-Wallis rank tests when not. *P* values of <0.05 were considered statistically significant. Statistical analysis was performed using Stata (v 14.0) software, and figures were generated using GraphPad Prism (v 8).

### Data availability.

All sequence data have been uploaded to the NCBI SRA database (SRP340092) and novel plasmids have been uploaded to the GenBank under OM975890 to OM975894.

10.1128/msphere.00143-22.10 DATA SET S1 The proportion of mapping depth (reference genome SWU01) of ≥10 samples. Download Data Set S1, XLSX file, 0.01 MB. Copyright © 2022 Zhang et al. 2022 Zhang et al. https://creativecommons.org/licenses/by/4.0/ This content is distributed under the terms of the Creative Commons Attribution 4.0 International license.
