# Supplementary material for: Sequence-Based Genomic Analysis Reveals Transmission of Antibiotic Resistance and Virulence among Carbapenemase-Producing Klebsiella pneumoniae Strains
Source: mSphere. 2022 May 12;7(3):e00143-22. doi: 10.1128/msphere.00143-22 (PMC9241541; doi:10.1128/msphere.00143-22)
Supplement: TABLE S1 [file msphere.00143-22-st001.docx]

**Supplementary Table 1. *Analysis of patients with more than one CP-Kpn isolates***

| Mono-place | Patient number | ST | Isolation Source | Gap time | SNP distance with the earliest isolate | Classification |
| --- | --- | --- | --- | --- | --- | --- |
| 8 | 1 | ST11 | sputum | - |  |  |
|  |  | ST11 | sputum | 14 | 6 | Within-host evolution |
|  | 2 | ST11 | sputum | - |  |  |
|  |  | ST11 | sputum | 7 | 0 | Within-host evolution |
|  | 3 | ST11 | sputum | - |  |  |
|  |  | ST11 | sputum | 14 | 0 | Within-host evolution |
|  | 4 | ST11 | sputum | - |  |  |
|  |  | ST11 | sputum | 15 | 18 | Within-host evolution |
|  | 5 | ST15 | sputum | - |  |  |
|  |  | ST15 | sputum | 2 | 72 | Mixed Infection |
|  | 6 | ST15 | sputum | - |  |  |
|  |  | ST15 | sputum | 29 | 946 | Mixed Infection |
|  | 7 | ST11 | sputum | - |  |  |
|  |  | ST11 | sputum | 17 | 2 | Within-host evolution |
|  | 8 | ST11 | sputum | - |  |  |
|  |  | ST11 | sputum | 16 | 6 | Within-host evolution |
|  | 9 | ST11 | Focal specimen | - |  |  |
|  |  | ST11 | Focal specimen | 5 | 31 | Mixed Infection |
|  |  |  |  |  |  |  |
| Multiple-places | 10 | ST11 | sputum | - |  | Mixed Infection |
|  |  | ST11 | Focal specimen | 5 | 0 |  |
|  |  | ST11 | sputum | 13 | 51 |  |
|  | 11 | ST11 | sputum | - |  |  |
|  |  | ST11 | CSF | 18 | 0 | Within-host evolution |
|  | 12 | ST11 | sputum | - |  |  |
|  |  | ST11 | Blood | 4 | 3 | Within-host evolution |
|  | 13 | ST11 | urine | - |  |  |
|  |  | ST11 | sputum | 5 | 1 | Within-host evolution |
|  | 14 | ST11 | Focal specimen | - |  |  |
|  |  | ST11 | sputum | 0 | 0 | Within-host evolution |
|  | 15 | ST11 | sputum | - |  |  |
|  |  | ST11 | urine | 24 | 2 | Within-host evolution |
|  | 16 | ST11 | sputum | - |  |  |
|  |  | ST11 | urine | 18 | 6 | Within-host evolution |
|  |  | ST11 | sputum | 19 | 6 | Within-host evolution |
|  | 17 | ST11 | Blood | - |  |  |
|  |  | ST11 | sputum | 6 | 8 | Within-host evolution |
|  | 18 | ST11 | urine | - |  |  |
|  |  | ST11 | Focal specimen | 7 | 2 | Within-host evolution |
|  | 19 | ST11 | urine | - |  |  |
|  |  | ST11 | Pharyngeal swab | 0 | 1 | Within-host evolution |
|  | 20 | ST11 | sputum | - |  |  |
|  |  | ST11 | urine | 29 | 4 | Within-host evolution |
|  | 21 | ST11 | urine | - |  |  |
|  |  | ST11 | sputum | 59 | 3 | Within-host evolution |
|  | 22 | ST11 | urine | - |  |  |
|  |  | ST11 | sputum | 1 | 1177 | Mixed Infection |
|  | 23 | ST11 | CSF | - |  |  |
|  |  | ST11 | sputum | 2 | 4 | Within-host evolution |
|  |  | ST11 | Catheter | 3 | 2 | Within-host evolution |
|  | 24 | ST11 | Blood | - |  | Mixed Infection |
|  |  | ST11 | sputum | 2 | 33 |  |
|  |  | ST11 | BALF | 2 | 33 |  |
|  |  | ST11 | sputum | 28 | 111 |  |
|  | 25 | ST11 | Tissue | - |  |  |
|  |  | ST11 | Focal specimen | 1 | 7 | Within-host evolution |
|  |  | ST11 | Blood | 2 | 7 | Within-host evolution |
|  |  | ST11 | Focal specimen | 7 | 11 | Within-host evolution |
| Different ST |  |  |  |  |  |  |
|  | 26 | ST11 | urine |  |  |  |
|  |  | ST15 | sputum |  |  |  |
|  | 27 | ST11 | Focal specimen |  |  |  |
|  |  | ST15 | sputum |  |  |  |
|  | 28 | ST11 | sputum | - |  | Mixed Infection & Within-host evolution |
|  |  | ST11 | sputum | 15 | 1 |  |
|  |  | ST437 | Catheter | - |  |  |
|  |  | ST437 | sputum | 5 | 1 |  |
