# Supplementary material for: Sequence-Based Genomic Analysis Reveals Transmission of Antibiotic Resistance and Virulence among Carbapenemase-Producing Klebsiella pneumoniae Strains
Source: mSphere. 2022 May 12;7(3):e00143-22. doi: 10.1128/msphere.00143-22 (PMC9241541; doi:10.1128/msphere.00143-22)
Supplement: TABLE S2 [file msphere.00143-22-st002.docx]

**Supplementary Table 2.** Antimicrobial Resistance profile of traditional AST and whole genome sequencing based identification

| **Antibiotic** | **Whole-genome Sequencing** | **AST** | | |
| --- | --- | --- | --- | --- |
|  | Resistance rate detected by WGS | AST Drugs performed | Resistance rate detected by AST | |
| **Aminoglycosides** |  |  | |  |
|  | 97.69% (169/173) | Amikacin | | 76.88% (133/173) |
|  |  | Gentamicin | | 86.13% (149/173) |
| **Colistin** |  |  | |  |
|  | 0.00% (0/173) | Not done | | / |
| **Fosfomycin** |  |  | |  |
|  | 66.47% (115/173) | Not done | | / |
| **Fluoroquinolone** |  |  | |  |
|  | 98.27% (170/173) | Ciprofloxacin | | 97/69% (169/173) |
| **Macrolides** |  |  | |  |
|  | 32.95% (57/173) | Not done | | / |
| **Sulfonamides** |  |  | |  |
|  | 61.27% (106/173) | SXT (compound SMZ) | | 66.47% (115/173) |
| **Tetracyclines** |  |  | |  |
|  | 64.74% (112/173) | Tigecycline | | 3.47% (6/173) |
| **Tyrimethoprim** |  |  | |  |
|  | 64.16% (111/173) | Not done | | / |
|  |  |  | |  |
| **Beta-lactamases** |  |  | |  |
|  | Extended spectrum beta-lactamases（ESBL）92.49%(160/173) | Cephalosporin | | 100.00% (173/173) |
|  | Carbapenems：100.00%(173/173) | Cefazolin | | 100.00% (173/173) |
|  |  | Cefuroxime | | 100.00% (173/173) |
|  |  | Cefotaxime | | 100.00% (173/173) |
|  |  | Ceftazidime | | 100.00% (173/173) |
|  |  | Cefepime | | 100.00% (173/173) |
|  |  | Piperacillin/tazobactam | | 100.00% (173/173) |
|  |  | Meropenem | | 99.42% (172/173) |
|  |  | Imipenem | | 97.69% (169/173) |
|  |  | Cefoperazone/sulbactam | | 98.84% (171/173) |
