# Supplementary figures and images for: Sequence-Based Genomic Analysis Reveals Transmission of Antibiotic Resistance and Virulence among Carbapenemase-Producing Klebsiella pneumoniae Strains
Source: mSphere. 2022 May 12;7(3):e00143-22. doi: 10.1128/msphere.00143-22 (PMC9241541; doi:10.1128/msphere.00143-22)

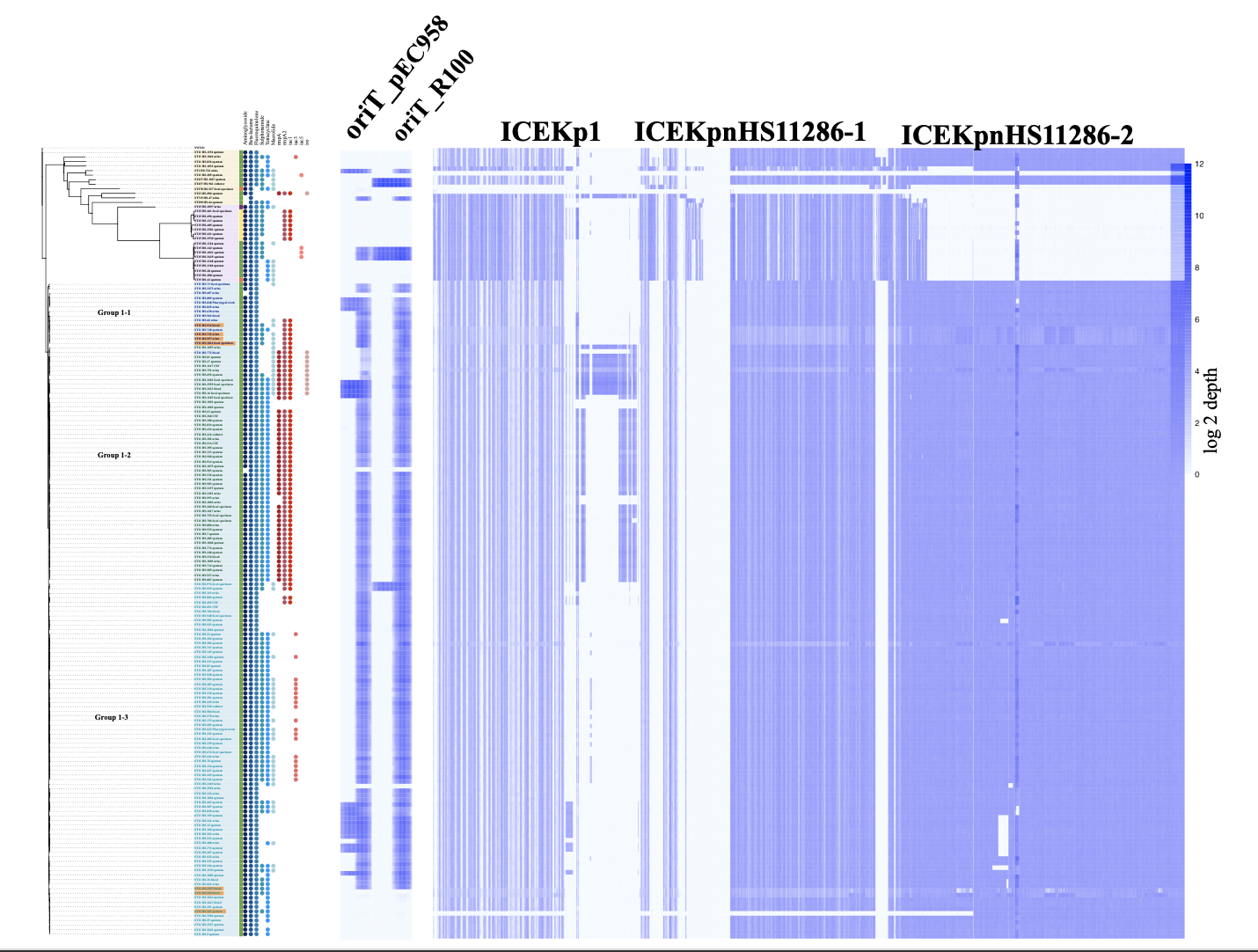

Supplement: FIG S1 [file msphere.00143-22-sf001.tif]

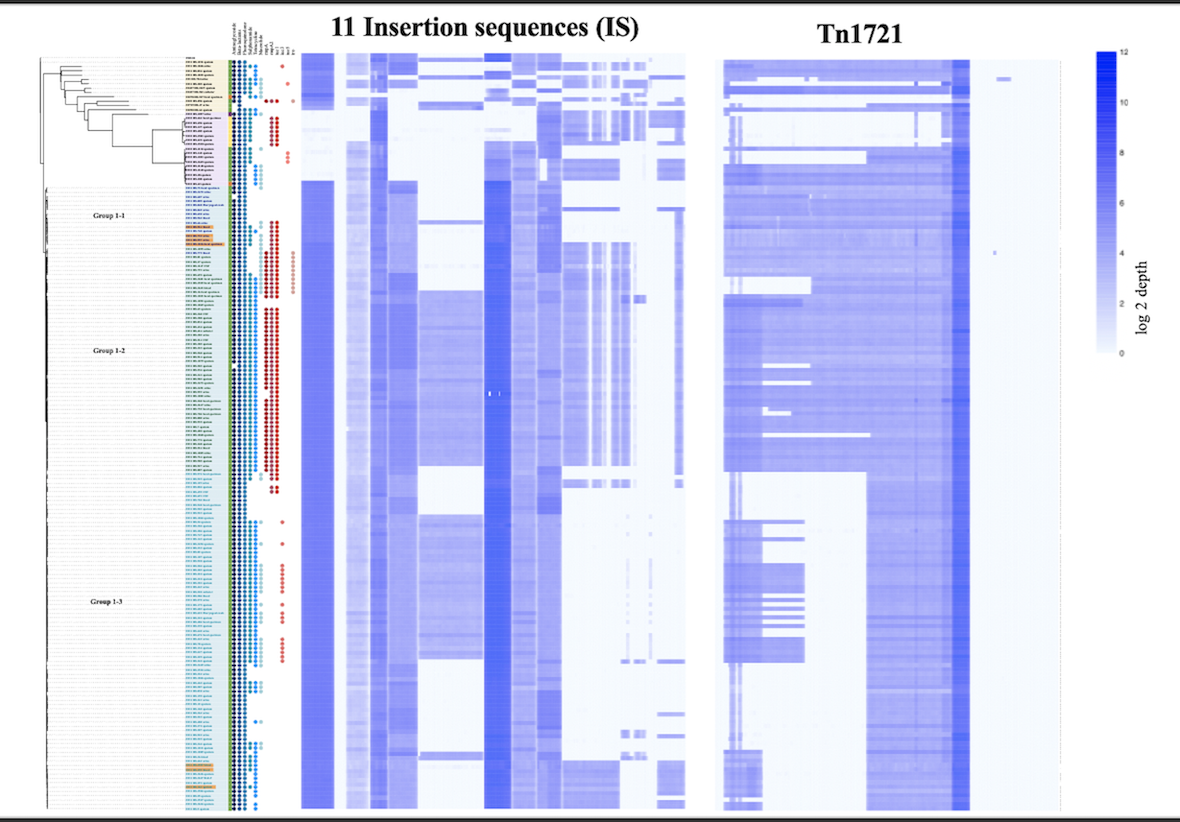

Supplement: FIG S2 [file msphere.00143-22-sf002.tif]

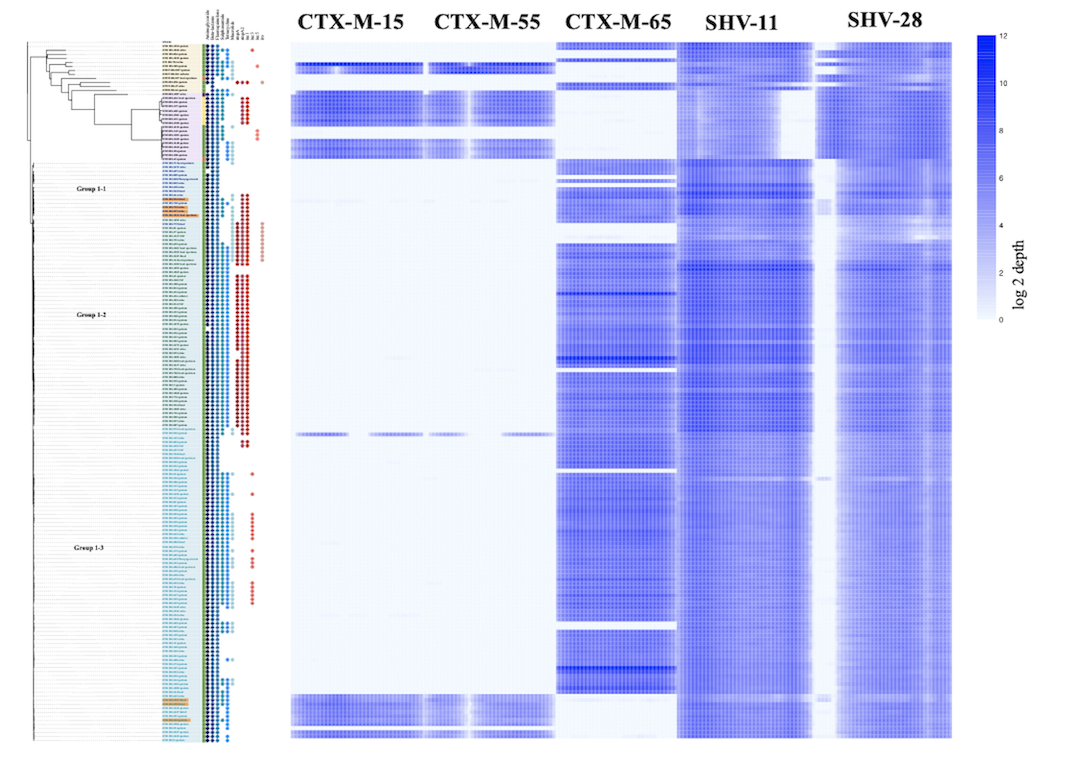

Supplement: FIG S3 [file msphere.00143-22-sf003.tif]

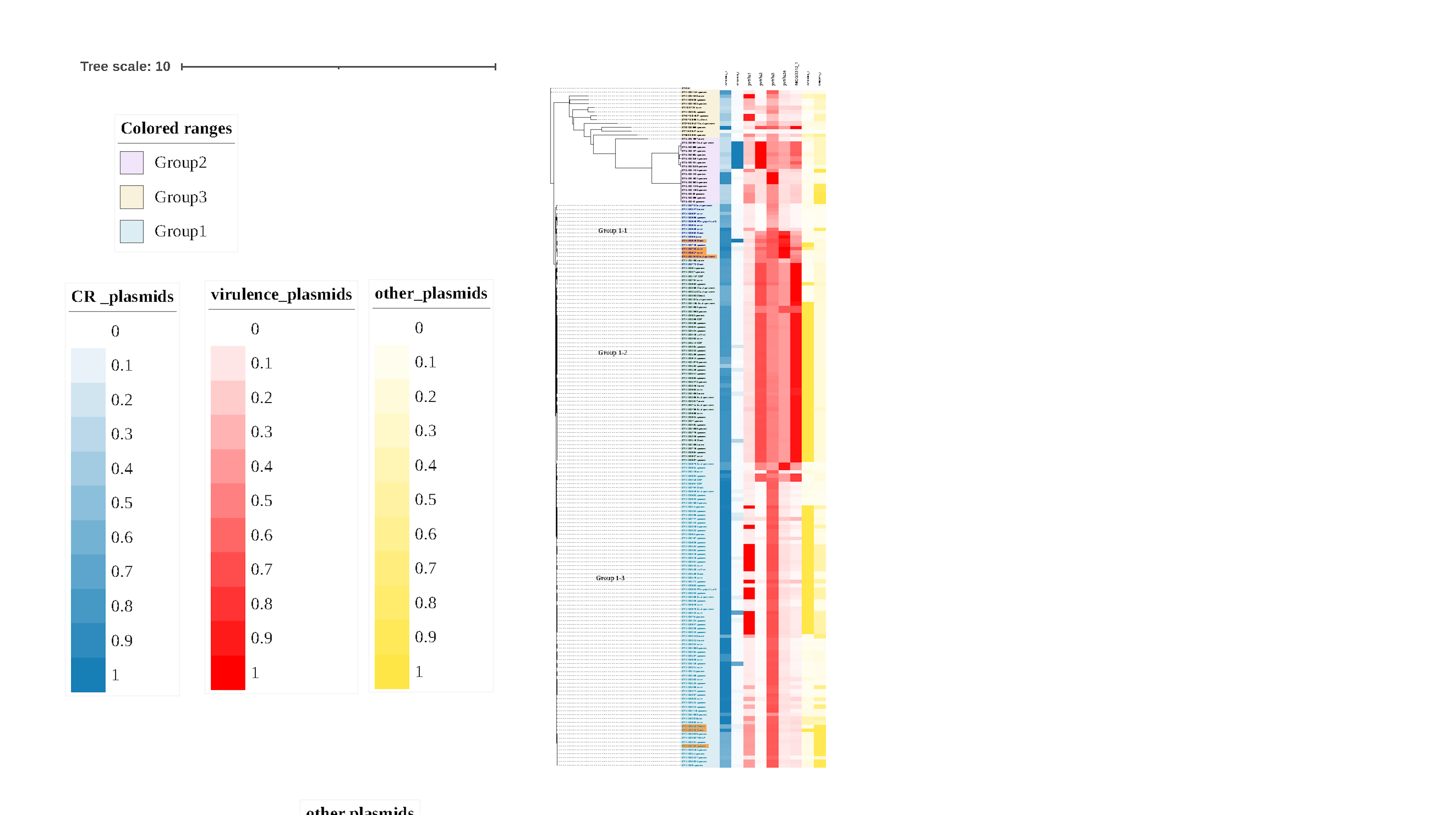

Supplement: FIG S4 [file msphere.00143-22-sf004.tif]

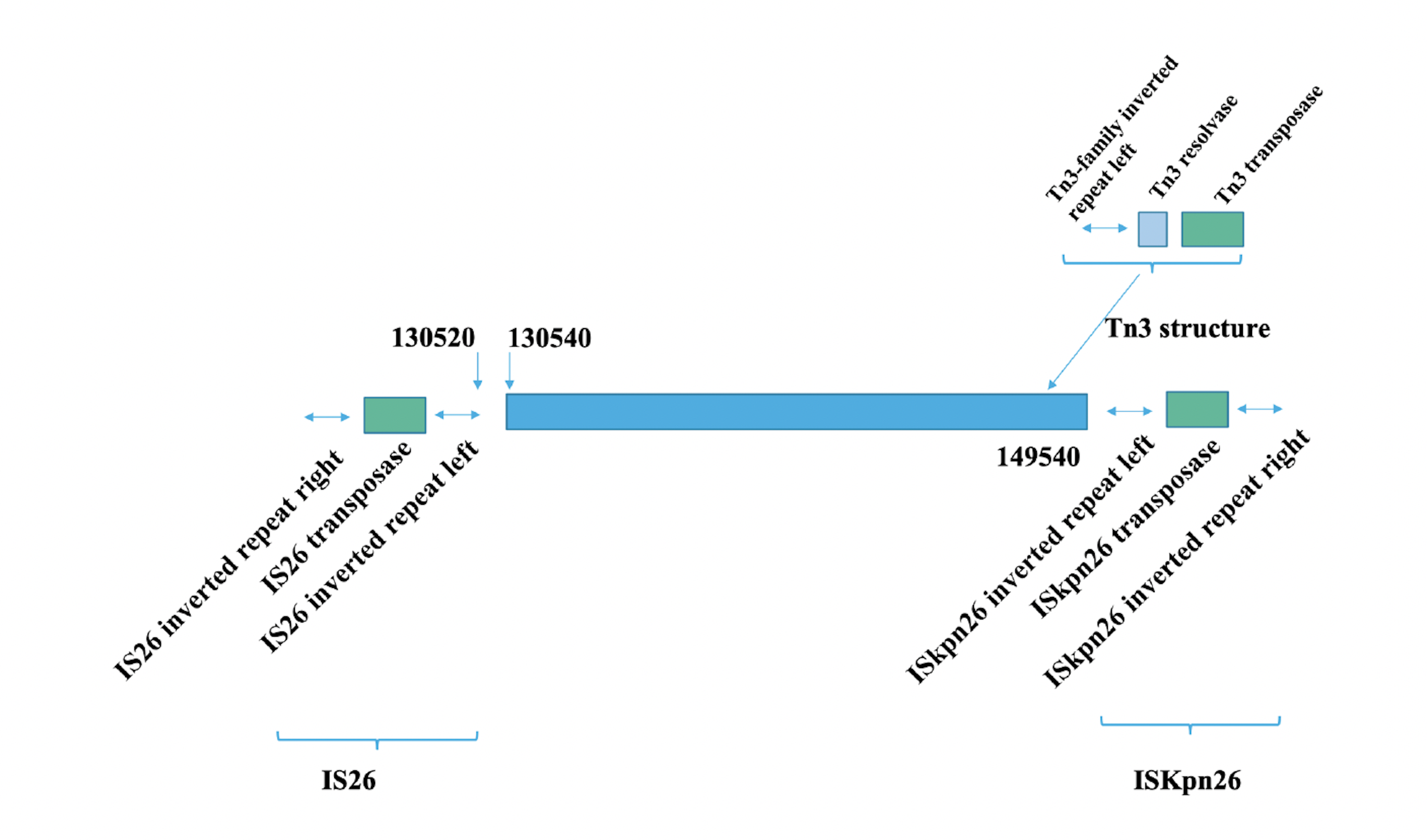

Supplement: FIG S5 [file msphere.00143-22-sf005.tif]
