# Supplementary material for: Sequence-Based Genomic Analysis Reveals Transmission of Antibiotic Resistance and Virulence among Carbapenemase-Producing Klebsiella pneumoniae Strains
Source: mSphere. 2022 May 12;7(3):e00143-22. doi: 10.1128/msphere.00143-22 (PMC9241541; doi:10.1128/msphere.00143-22)
Supplement: TABLE S3 [file msphere.00143-22-st003.docx]

**Supplementary Table 3. The virulence score ranges from 0 to 5 scores^1,2^.**

| **score** |  |  |
| --- | --- | --- |
| 0 | none of the acquired virulence loci (i.e. negative for all of yersiniabactin, colibactin, aerobactin, salmochelin) |  |
| 1 | yersiniabactin only |  |
| 2 | yersiniabactin and colibactin, or colibactin only |  |
| 3 | aerobactin and/or salmochelin only (without yersiniabactin or colibactin) |  |
| 4 | aerobactin and/or salmochelin with yersiniabactin (without colibactin) |  |
| 5 | yersiniabactin, colibactin and aerobactin and/or salmochelin |  |
|  |  | |

**Reference:**

**1.** Lam MMC, Wyres KL, Judd LM, et al. Tracking key virulence loci encoding aerobactin and salmochelin siderophore synthesis in Klebsiella pneumoniae. *Genome Med* 2018; **10**(1): 77.

2. https://github.com/katholt/Kleborate.
