# Supplementary material for: Sequence-Based Genomic Analysis Reveals Transmission of Antibiotic Resistance and Virulence among Carbapenemase-Producing Klebsiella pneumoniae Strains
Source: mSphere. 2022 May 12;7(3):e00143-22. doi: 10.1128/msphere.00143-22 (PMC9241541; doi:10.1128/msphere.00143-22)
Supplement: TABLE S4 [file msphere.00143-22-st004.docx]

**Supplementary Table 4. List of isolates sequenced by illumina and Nanopore and aligned plasmid**

| Number | Isolate Number | Plasmid 1 (Coverage) | Plasmid 2 (Coverage) | Plasmid 3 (Coverage) | Plasmid 4 (Coverage) | Plasmid 5 (Coverage) | Plasmid 6 (Coverage) |
| --- | --- | --- | --- | --- | --- | --- | --- |
| 1 | HS-3597 | CP002474 (94.14%) | MF133495 (62.19%) | CP025459 (100.00%) |  |  |  |
| 2 | HS-732 | pvirhs 4  (OM975894) | CP006799 (79.84%) | MF133495 (95.49%) | CP025459 (100.00%) | CP023936 (100.00%) |  |
| 3 | HS-692 | MG053312 (100.00%) | FJ876827（64.00%） | MF133495（80.25%） | CP025459 (100.00%) |  |  |
| 4 | HS-9HS | MF133495 (88.20%) | CP025459 (100.00%) | CP023936 (100.00%) |  |  |  |
| 5 | HS-3494 | pvirhs 1  (OM975891) | MF133495 (99.93%) | MF133496 (100.00%) | CP025459 (100.00%) | CP023936 (100.00%) |  |
| 6 | HS-3501 | pvirhs 2  (OM975892) | JX424423 (90.09%) | CP008931 (100.00%) | LN824135 (56.90%) | CP006658  (51.37%) | KY454616  (99.98%) |
| 7 | HS-63 | MG053312 (93.66%) | MF133495 (80.05%) | MF133496 (100.00%) | CP025459 (100.00%) | CP023936 (100.00%) |  |
| 8 | HS-78 | pvirhs 1  (OM975891) | MF133495 (99.94%) | MF133496 (100.00%) | CP025459 (100.00%) | CP023936 (100.00%) |  |
| 9 | HS-142 | pvirhs 3  (OM975893) | MF133495 (87.38%) | KX236178 (54.68%) | CP025459 (100.00%) | CP023936 (100.00%) |  |
| 10 | HS-1097 | HF545434  (82.64%) | phs-IMP8  (OM975890) | CP025459 (100.00%) |  |  |  |
| 11 | HS-527 | CP011623 (28.48%) | MF437312 (31.74%) | KF220657 (100.00%) | EU383016 (84.06%) |  |  |
| 12 | HS-7 | MG053312 (93.66%) | MF168404 (97.69%) | MF144193 (89.04%) | MF133496 (100.00%) | CP023932  (100.00%) | CP023936 (100.00%) |
| 13 | HS-12 | MF133495 (99.96%) | FJ876827 (81.20%) | CP025459 (100.00%) | CP023936 (100.00%) |  |  |
